# Breastfeeding self-efficacy in mothers in socioeconomic vulnerability: Situation-Specific Theory

**DOI:** 10.1590/0034-7167-2024-0652

**Published:** 2025-12-08

**Authors:** Rebecca Camurça Torquato, Joana Maria Rocha Sales, Laysla de Oliveira Cavalcante Lima, Marcos Venícios de Oliveira Lopes, Viviane Martins da Silva, Lorena Pinheiro Barbosa

**Affiliations:** I Universidade Federal do Ceará. Fortaleza, Ceará, Brazil

**Keywords:** Self Efficacy, Nursing Theory, Mothers, Breast Feeding, Socioeconomic Factors., Autoeficacia, Teoría de Enfermería, Madres, Lactancia Materna, Factores Socioeconómicos.

## Abstract

**Objectives:**

to develop a Situation-Specific Theory based on the Self-Efficacy Theory applied to breastfeeding mothers in socioeconomic vulnerability.

**Methods:**

the Situation-Specific Theory was constructed based on Callista Roy’s assumptions, structured in six stages: (1) defining approach; (2) defining conceptual models; (3) defining the main concept; (4) developing a pictorial diagram; (5) constructing a proposition; (6) establishing causal relationships and evidence for practice.

**Results:**

eight socioeconomic factors such as age, parity, maternal occupation, education, marital status, race, social support and family income interfere with the sources of self-efficacy in mothers in socioeconomic vulnerability. The Situation-Specific Theory elements were associated in a pictorial diagram and in causal relationships evidenced by six propositions.

**Conclusions:**

it is concluded that the Situation-Specific Theory can provide a better understanding of the relationships between these factors with the sources of Self-Efficacy Theory in mothers with socioeconomic vulnerability.

## RESUMEN

**Objetivos:**

desarrollar una Teoría de la Situación Específica basada en la Teoría de la Autoeficacia aplicada a madres lactantes en situación de vulnerabilidad socioeconómica.

**Métodos:**

la Teoría de la Situación Específica se construyó con base en los supuestos de Callista Roy, estructurada en seis etapas: (1) definición del enfoque; (2) definición de los modelos conceptuales; (3) definición de los conceptos principales; (4) desarrollo del diagrama pictórico; (5) construcción de proposiciones; (6) establecimiento de relaciones causales y evidencia para la práctica.

**Resultados:**

ocho factores socioeconómicos, como edad, paridad, ocupación materna, educación, estado civil, raza, apoyo social e ingreso familiar, interfieren en las fuentes de autoeficacia en madres en vulnerabilidad socioeconómica. Los elementos de la Teoría de la Situación Específica se asociaron en un diagrama pictórico y en relaciones causales evidenciadas por seis proposiciones.

**Conclusiones:**

se concluye que la Teoría de la Situación Específica puede proporcionar una mejor comprensión de las relaciones entre estos factores y las fuentes de la Teoría de la Autoeficacia en madres con vulnerabilidad socioeconómica.

## INTRODUCTION

Nursing theories favor theoretical development, contributing to quality care and enabling nursing to structure its professional and scientific field^(1)^. From this perspective, theories may not be applicable to all social structures, political situations or historical periods^(2)^, making it essential to develop theoretical frameworks that are appropriate to the population in question and their needs.

Theories can be defined as the set of assumptions, principles or propositions that project a systematic view of phenomena extracted from reality, offering the organization and structure of knowledge^(3)^. These theories can vary according to the level of scope so that grand theories are broad, while Middle-Range Theory (MRT) and Situation-Specific Theory (SST) are more specific^(4)^.

In the context of grand theories, Bandura’s Self-Efficacy Theory^(5)^ can be cited. This theory presents self-efficacy as a set of different self-beliefs related to domains of different functioning^(5)^. Such self-efficacy beliefs are developed based on four essential sources of information, namely: enactive mastery experience, which function as parameters of capacity; vicarious experience, which modify the conviction of efficacy based on the observation of models of others; verbal persuasion, in which the belief of self-efficacy is reinforced through others’ social influence; and emotional and physiological state, from which individuals judge their competence, strength and fragility^(5)^.

MRTs, being more specific, present fewer concepts and involve a more limited scope of reality, with concrete and operationally defined propositions^(6)^. Grand theories and MRTs, however, have limitations in portraying the diversity and description of specific phenomena in nursing^(7)^. Thus, in 1999, SSTs emerged, which constitute theories focused on specific populations or fields of practice, inserted in particular social and historical contexts, being considered more practical and appropriate for research in population groups when compared to MRTs^(7)^.

In 1999, nurse Cindy-Lee Dennis, recognizing the importance of the grand Theory of Self-Efficacy and following her enthusiasm for research involving breastfeeding and the World Health Organization recommendations on its promotion and encouragement for maternal and child health^(8)^, built a theoretical framework on self-efficacy in the context of breastfeeding. The author, in her writings, develops arguments that reinforce the applicability of Bandura’s theoretical assumptions to a specific reality, such as breastfeeding, by relating the four sources of self-efficacy to the specificities of a breastfeeding woman^(9)^.

In the context of breastfeeding, there is a gap in the development of SST aimed at mothers in situations that are unfavorable to the breastfeeding process. Among these situations, socioeconomic vulnerability stands out as a condition in which affected individuals are less likely to face difficult economic situations and believe they are not entitled to benefits that could help protect them from such deprivation^(10)^. When citing that these conditions influence breastfeeding, the question is: how can we explain the phenomenon of breastfeeding self-efficacy in mothers in situations of socioeconomic vulnerability?

The construction of an SST for this specific audience can contribute to a better understanding of how breastfeeding self-efficacy presents itself in this context and, thus, provide the framework for planning care directed at this woman, aiming at promoting breastfeeding and the health of the mother-baby dyad.

## OBJECTIVES

To develop an SST based on the Self-Efficacy Theory applied to breastfeeding mothers in socioeconomic vulnerability.

## METHODS

This study followed six stages: defining the approach to SST; defining conceptual models; defining the main concepts; developing a pictorial diagram; constructing propositions; establishing causal relationships and evidence for practice^(11)^.

### Defining the approach to the Situation-Specific Theory

In search of a scientific and reflective basis for SST construction, Bandura’s Self-Efficacy Theory^(5)^ and Callista Roy’s theoretical assumptions were used as a basis^(11)^, a scoping review was carried out with a protocol registered in the Open Science Framework (https://doi.org/10.17605/OSF.IO/CFTA7). This was reported according to the Preferred Reporting Items for Systematic Reviews and Meta-Analyses for Scoping Reviews (PRISMA-ScR)^(12)^, following the methodological recommendations proposed by the JBI for scoping reviews^(13)^.

The review was carried out during the months of May to June 2024. The guiding question was based on the Population, Concept, Context (PCC) acronym indicated by the JBI^(13)^, in which “P” includes breastfeeding mothers, “C”, self-efficacy in breastfeeding, and “C”, socioeconomic vulnerability, which generated the following question: what are the elements and relationships that influence self-efficacy in breastfeeding in the population of breastfeeding mothers in socioeconomic vulnerability? Chart 1 summarizes the stages performed.

**Chart 1:** Research question and construction of the scoping review search strategy, Fortaleza, Ceará, Brazil, 2024

Search question	What are the elements and relationships that influence breastfeeding self-efficacy in the population of breastfeeding mothers in socioeconomic vulnerability?		
	Population	Concept	Context
**Extraction**	Breastfeeding mothers	Breastfeeding self-efficacy	Socioeconomic vulnerability
**Conversion**	Breastfeeding	Self-efficacy	Socioeconomic factors
**Combination**	breastfeeding; breastfeeding; supplemented breastfeeding; exclusive breastfeeding; breastfed; breastfeeding; breast fed; breast feeding, exclusive; exclusive breast feeding; breastfeeding, exclusive; exclusive breastfeeding; complementary feeding; feeding, complementary; breast feeding education; breast feeding promotion; breast feeding support; breastfeeding education; breastfeeding promotion; breastfeeding support	self-efficacy; self efficacy; efficacy, self; self concept; concept, self; self; self awareness; self efficacy; self confrontation; self image; self perception; self rating; self representation; self concept	socioeconomic factors; economic and social factors; socioeconomic characteristics; social inequity; socioeconomic vulnerability; factor, socioeconomic; socioeconomic factors; factors, socioeconomic; social inequalities; socioeconomic factor; economic and social factors; social inequality; social and economic factors; socioeconomic vulnerability; socioeconomics; economic value of life; socioeconomic characteristics; characteristic, socioeconomic; health care, indigent; inequality, social; medical indigency; socioeconomic characteristic; indigent health care; social economic aspect; social economics; social-economic factor; socio-economic aspect; socio-economic factor; socio-economics; socioeconomic aspect; value of life; socioeconomics; social economic vulnerability; socio-economic vulnerability
**Construction**	breastfed OR breastfeeding OR “breast fed” OR “breast feeding, exclusive” OR “exclusive breast feeding” OR “breastfeeding, exclusive” OR “exclusive breastfeeding” OR “complementary feeding” OR “feeding, complementary” OR “breast feeding education” OR “breast feeding promotion” OR “breast feeding support” OR “breastfeeding education” OR “breastfeeding promotion” OR “breastfeeding support” OR “breast feeding education”	“self efficacy” OR “efficacy, self” OR “self concept” OR “concept, self” OR self OR “self awareness” OR “self confrontation” OR “self efficacy” OR “self image” OR “self perception” OR “self rating” OR “self representation” OR selfconcept	“socioeconomic factors” OR “factor, socioeconomic” OR “socioeconomic factor” OR “economic and social factors” OR “factors, socioeconomic” OR “socioeconomic characteristics” OR “characteristic, socioeconomic” OR “socioeconomic characteristic” OR “social and economic factors” OR “social inequality” OR “inequality, social” OR “social inequalities” OR “socioeconomic vulnerability” OR socioeconomics OR “economic value of life” OR “health care, indigent” OR “indigent health care” OR “medical indigency” OR “social economic aspect” OR “social economics” OR “social-economic factor” OR “socio-economic aspect” OR “socio-economic factor” OR “socio-economics” OR “socioeconomic aspect” OR “value of life” OR “social economic vulnerability” OR “socio-economic vulnerability”
**Main search strategy**	((breastfed OR breastfeeding OR “breast fed” OR “breast feeding, exclusive” OR “exclusive breast feeding” OR “breastfeeding, exclusive” OR “exclusive breastfeeding” OR “complementary feeding” OR “feeding, complementary”) AND (“self efficacy” OR “efficacy, self”)) AND (“socioeconomic factors” OR “factor, socioeconomic” OR “socioeconomic factor” OR “economic and social factors” OR “factors, socioeconomic” OR “socioeconomic characteristics” OR “characteristic, socioeconomic” OR “socioeconomic characteristic” OR “social and economic factors” OR “social inequality” OR “inequality, social” OR “social inequalities” OR “socioeconomic vulnerability”)		

*Source: adapted from Araújo OWC, 2020^(14)^.*

Based on the guiding question, a search was conducted in the MEDLINE (via PubMed), Excerpta Medica Database (Embase), Latin American and Caribbean Literature in Health Sciences (LILACS), Cumulative Index to Nursing and Allied Health Literature via EBSCO (CINAHL), Scopus, Web of Science and Cochrane Library databases.

**Figure 1:**
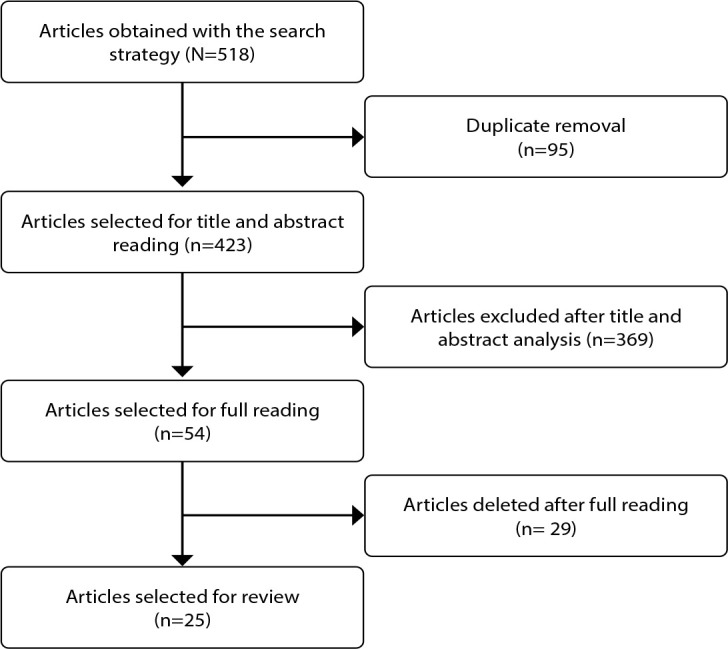
Preferred Reporting Items for Systematic Reviews and Meta-Analyses for Scoping Reviews diagram of search and selection of articles for the scoping review, Fortaleza, Ceará, Brazil, 2024

Studies of all methodological designs, available in full, in English, Portuguese and Spanish, with no restriction on publication date, were included. Articles that did not answer the research question, as well as reviews, editorials, books, chapters, opinion pieces, dissertations, theses and conference abstracts due to the flexibility of the assessment criteria for these productions, and articles not available in full were excluded. The phases involved in the process of selecting the analyzed articles are described in the PRISMA-ScR diagram (Figure 1).

The Rayyan software was used to manage the productions, and the articles were assessed and selected based on eligibility criteria by two independent and blind reviewers. Any discrepancies were resolved by a third reviewer.

Data from the selected studies were extracted and analyzed by completing a characterization form based on the JBI guidelines^(13)^ and structured with the following data: (1) study characteristics: title, year, country, journal, authors, study design, objectives; (2) population characteristics: sample size, sociodemographic data, general characteristics and socioeconomic vulnerability; (3) main outcome: breastfeeding self-efficacy and related socioeconomic factors.

### Defining conceptual models

To construct the SST, Bandura’s Self-Efficacy Theory^(5)^ conceptual model was used to construct the concepts, relationships and assumptions reported in this study. This defines self-efficacy as the confidence that individuals have the ability to successfully perform certain activities or behaviors. This conviction is constructed by four main sources of self-efficacy, which are enactive mastery experience, vicarious experience, verbal persuasion, and emotional and physiological state.

### Defining the main concepts

According to evidence from the scoping review and Bandura’s Self-Efficacy Theory assumptions^(5)^, key concepts were selected to compose the minimum elements for constructing an SST: self-efficacy in breastfeeding; enactive mastery experience; vicarious experience; verbal persuasion; physiological and affective state; and socioeconomic vulnerability.

### Developing a pictorial diagram

The pictorial diagram of this study was developed from the primary and secondary information of the structuring concepts of SST, enabling the understanding of causal relationships.

### Constructing propositions

To explain the relationships between concepts within SST, six propositions were developed to demonstrate the relationships between socioeconomic factors and breastfeeding self-efficacy in mothers in socioeconomic vulnerability, stratified into Bandura’s four sources of self-efficacy.

### Establishing causal relationships and evidence for practice

The causal relationships between the SST elements were expressed within the pictorial diagram, but these may not be visualized in their entirety. Thus, these relationships between socioeconomic elements and breastfeeding self-efficacy in mothers in socioeconomic vulnerability were also described through propositions to facilitate their understanding and use of SST in clinical practice.

## RESULTS

Chart 2 describes the articles selected to provide the theoretical basis, together with the Self-Efficacy Theory^(5)^, for developing an SST, their authors, year of publication and study design.

**Chart 2:** Description of articles included in the scoping review, Fortaleza, Ceará, Brazil, 2024

References	Year Country	Title	Study design	Level of evidence^*^
Siqueira *et al*.^(15)^	2023 Brazil	Factors associated with breastfeeding self-efficacy in the immediate puerperium in a public maternity hospital	Cross-sectional study	Level 6
Pinto *et al*.^(16)^	2021 Brazil	Evaluation of breastfeeding self-effectiveness and its associated factors in puerperal women assisted at a public health system in Brazil	Cross-sectional study	Level 6
Müller *et al*.^(17)^	2020 Brazil	Self-efficacy and exclusive breastfeeding maintenance in the first months after childbirth	Prospective cohort study	Level 4
Santos *et al*.^(18)^	2020 Brazil	Self-efficacy of breastfeeding in postpartum women assisted in a public maternity in northeastern Brazil	Cross-sectional study	Level 6
Monteiro *et al*.^(19)^	2020 Brazil	Breastfeeding self-efficacy in adult women and its relationship with exclusive maternal breastfeeding	Longitudinal and prospective study	Level 4
Silva *et al*.^(20)^	2018 Brazil	Breastfeeding self-efficacy and interrelated factors	Cross-sectional study	Level 6
Guimarães *et al*.^(21)^	2017 Brazil	Factors related with breastfeeding self-efficacy immediate after birth in puerperal adolescents	Cross-sectional study	Level 6
Santos *et al*.^(22)^	2022 Brazil	Self-efficacy of puerperal women in breastfeeding: a longitudinal study	Longitudinal panel study	Level 4
Rodrigues *et al*.^(23)^	2015 Brazil	Influence of sociodemographic and behavioral conditions on self-efficacy in breastfeeding: a cross-sectional study	Cross-sectional study	Level 6
Uchôa *et al*.^(24)^	2014 Brazil	Sociodemographic and obstetric history in maternal self-efficacy in nursing: a study in panel	Longitudinal panel study	Level 4
Margotti and Epifanio^(25)^	2014 Brazil	Exclusive maternal breastfeeding and the Breastfeeding Self-efficacy Scale	Descriptive and analytical cohort study	Level 4
Nursan, Dilek and Sevin^(26)^	2014 Turkey	Breastfeeding Self-efficacy of Mothers and the Affecting Factors	Descriptive study	Level 6
Dodt *et al*.^(27)^	2013 Brazil	Influence of health education strategy mediated by a self-efficacy breastfeeding serial album	Quasi-experimental study	Level 3
Quiroz *et al*.^(28)^	2013 Peru	*Lactancia materna exitosa en puérperas de menos de 48 horas en el Hospital de Apoyo María Auxiliadora*	Cross-sectional study	Level 6
Al-Thubaity *et al.* ^(1)^	2023 Saudi Arabia	Determinants of High Breastfeeding Self-Efficacy among Nursing Mothers in Najran, Saudi Arabia	Cross-sectional study	Level 6
Li *et al.* ^(29)^	2022 China	Determinants of breastfeeding self-efficacy among postpartum women in rural China: A cross-sectional study	Cross-sectional study	Level 6
Yang *et al*.^(30)^	2016 China	Predictors of breastfeeding self-efficacy in the immediate postpartum period: A cross-sectional study	Cross-sectional study	Level 6
Tavares *et al*.^(31)^	2010 Brazil	Application of Breastfeeding Self-Efficacy Scale-Short Form to post-partum women in rooming-in care: a descriptive study	Cross-sectional study	Level 6
Souza *et al*.^(32)^	2020 Brazil	*Avaliação da autoeficácia na amamentação em puérperas*	Epidemiological study	Level 6
Joshi *et al.* ^(33)^	2015 United States of America	Comparison of Socio-Demographic Characteristics of a Computer Based Breastfeeding Educational Intervention Among Rural Hispanic Women	Quasi-experimental study	Level 3
Ku and Chow^(34)^	2010 China	Factors influencing the practice of exclusive breastfeeding among Hong Kong Chinese women: a questionnaire survey	Cross-sectional study	Level 6
Kamalifard *et al*.^(35)^	2019 Iran	Relationship of Breastfeeding Self-Efficacy with Self-Esteem and General Health in Breastfeeding Mothers Referred to Health Centers of Falavarjan City-Iran, 2015	Cross-sectional study	Level 6
Gökçeoğlu and Küçükoğlu^(36)^	2016 Turkey	The relationship between insufficient milk perception and breastfeeding self-efficacy among Turkish mothers	Descriptive study	Level 6
Lima *et al*.^(37)^	2023 Brazil	*Avaliação do uso de álbum seriado sobre amamentação como estratégia de intervenção educativa no puerpério*	Quasi-experimental study	Level 3
Conde *et al*.^(38)^	2017 Brazil	Breastfeeding self-efficacy and length of exclusive breastfeeding among adolescent mothers	Prospective, observational and analytical longitudinal study	Level 4

* *Level of evidence-Melnyk; Fineout-Overholt, 2023^(39)^.*

### Situation-Specific Theory of breastfeeding self-efficacy in mothers in socioeconomic vulnerability

The SST development was based on the 25 articles identified in the review as well as on the Self-Efficacy Theory assumptions. Regarding the method, among the articles analyzed, 13 were cross-sectional studies, four were longitudinal studies, three were quasi-experimental studies, two were cohort studies, two were descriptive studies, and one was an epidemiological study.

### Socioeconomic factors

The socioeconomic factors identified in the scoping review that may influence breastfeeding self-efficacy were maternal age, family income, marital status, number of children, education, occupation, race, and social support.

The most frequent factor was the number of children, present in ten articles, followed by maternal age and family income, both reported in nine studies. Other relevant factors for breastfeeding self-efficacy in this population include education, cited in eight articles, marital status, cited in seven articles, social support, cited in six articles, and occupation, cited in five articles. Moreover, it was observed that race, cited in only one study, can also influence the object of study.

### Defining the main concepts

From the scoping review and the background literature, the main concepts for the SST in question were developed, namely:

- Breastfeeding self-efficacy: mothers’ perception of their ability and capacity to breastfeed their children;
- Active domain experience: previous personal experience in breastfeeding that helps mothers in their current breastfeeding practice;
- Vicarious experience: observation of an individual who has experienced the breastfeeding process, who can offer support to mothers by sharing their experiences;
- Verbal persuasion: transmission of knowledge through guidance and encouragement by an individual, which aims to encourage mothers to breastfeed;
- Emotional and physiological state: emotional and health condition in which mothers find themselves and which affects, positively or negatively, their breastfeeding process;
- Socioeconomic vulnerability: situation in which socioeconomic factors affect the breastfeeding process of mothers, making them more susceptible to having difficulties in establishing breastfeeding and maintaining adequate levels of self-efficacy in breastfeeding.

### Developing a pictorial diagram

**Figure 2:**
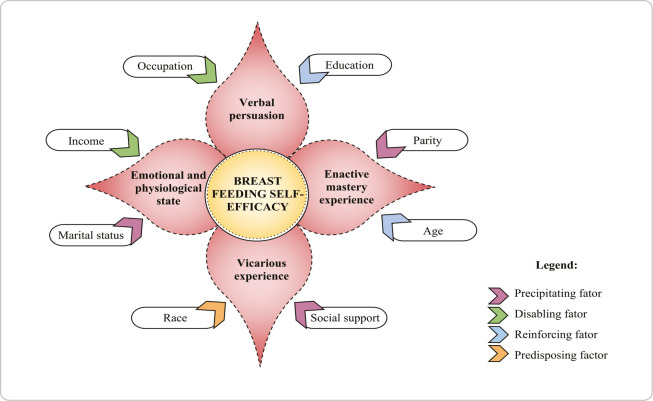
Pictogram representing the causal relationships of Situation-Specific Theory for breastfeeding self-efficacy in mothers in socioeconomic vulnerability, Fortaleza, Ceará, Brazil, 2024

After developing the main concepts, a pictorial diagram was constructed to aid in understanding SST (Figure 2).

### Constructing propositions

According to Fawcett^(40)^, propositions are true principles without the need for proof. For the SST of this study, the following propositions were developed:

1. Younger maternal age and, consequently, a greater chance of primiparity, are reinforcing and precipitating factors, respectively, that can lead to low levels of breastfeeding self-efficacy, since these women generally do not have experience in actively controlling breastfeeding;
2. Being single is a precipitating factor for low levels of breastfeeding self-efficacy, as the absence of a partner affects women’s emotional and physiological state;
3. Having a low income is a disabling factor that can negatively affect levels of self-efficacy in breastfeeding, as it affects mothers’ emotional and physiological state in a context of apprehension resulting from scarce financial resources;
4. Maintaining work activity outside the home, considered a disabling factor, may be related to lower levels of self-efficacy in breastfeeding, since mothers have limited access to information from verbal persuasion sufficient to maintain breastfeeding after returning to work;
5. Low education level is a reinforcing factor that can hinder mothers’ understanding of information arising from verbal persuasion activities, which undermines their self-efficacy in breastfeeding;
6. Maternal race, considered a predisposing factor, can influence levels of self-efficacy in breastfeeding, since ethnicity and social support, classified as precipitating factors, enable the sharing of vicarious experiences, encouraging continued breastfeeding.

### Establishing causal relationships and evidence for practice

The causal relationships between socioeconomic factors and breastfeeding self-efficacy are described in detail in a pictorial diagram and in propositions. The elements influence the levels of breastfeeding self-efficacy so that: social support, marital status and parity are considered precipitating factors; income and occupation are considered disabling factors; age and education are considered reinforcing factors; and race is considered a predisposing factor.

## DISCUSSION

The breastfeeding Self-Efficacy Theory in mothers in socioeconomic vulnerability explains essential concepts for the construction of knowledge about this phenomenon, in addition to listing the socioeconomic factors that intervene in the self-efficacy presented by mothers in this specific situation. Causal relationships between the main concepts of the constructed SST will be explained according to evidence extracted from the current literature.

The number of children, maternal age and income are the socioeconomic factors most frequently associated with breastfeeding self-efficacy in the analyzed clientele. Primiparity is a factor that predisposes to reduced breastfeeding self-efficacy^(1,15,17,18,22,23,25,28,30)^. This proposition is related to previous personal experiences in breastfeeding, common in multiparous mothers and absent in primiparous mothers, influencing one of the sources of self-efficacy pointed out by Bandura^(5)^ and highlighted by Dennis^(9)^ as fundamental for high self-efficacy in breastfeeding^(1)^.

Most evidence also shows that older maternal age is associated with higher levels of breastfeeding self-efficacy^(15,18,22,27,28,31)^. Maternal age and parity are, in turn, interrelated factors. Women who have their first child at a young age may have more births over the course of their lives^(41)^, which reflects previous breastfeeding experiences, increasing the levels of breastfeeding self-efficacy in these mothers^(1,42)^.

Although this relationship is well established, certain studies conducted in Brazil^(17-18)^ and China^(34)^ show that lower age groups have a relationship with higher breastfeeding self-efficacy due to the support provided by mothers/mothers-in-law, acting as sources of vicarious experience for younger mothers^(21)^.

Family income and breastfeeding self-efficacy in mothers in socioeconomic vulnerability also have a directly proportional relationship, and this could be observed in several studies in this review^(15,20,22,24,27,31,34)^. This relationship can be clarified by the fact that family income directly affects mothers’ emotional and physiological state, one of the sources of self-efficacy proposed by Bandura, as breastfeeding mothers worry about the family situation and this can affect their lactation process and, consequently, breastfeeding and the self-efficacy to put it into practice.

Rodrigues *et al*.^(23)^ and Kamalifard *et al*.^(35)^ validate this proposition by pointing out that the presence of four or more people living in the same household with a single source of income and the unemployment of spouses are factors associated with a reduced level of maternal self-efficacy in breastfeeding, since these conditions lead to unfavorable economic situations, affecting women’s emotional and physiological state, which leads to a decline in general health status concomitant with breastfeeding self-efficacy.

Still regarding family income and its impact on breastfeeding, a study conducted by Uchôa *et al*.^(24)^ found that the presence of basic sewage and water supply services positively influences the level of self-efficacy in breastfeeding in mothers in socioeconomic vulnerability. Low coverage of these services and inaccessibility to solid waste collection may be detrimental factors associated with self-perceived health^(43)^, affecting mothers’ emotional and physiological state, and feeding back into the status of low breastfeeding self-efficacy. Despite their importance, lowand middle-income countries lack waste management and access to sanitation and water supply, highlighting the need for better coverage in order to produce substantially beneficial effects on health^(44)^ as well as on mothers’ breastfeeding self-efficacy.

In mothers with socioeconomic vulnerability, marital status was also directly associated with breastfeeding self-efficacy. Support from husbands or partners is also considered a predictor of breastfeeding self-efficacy^(30)^ so that married mothers and those in consensual unions had higher levels of breastfeeding self-efficacy^(15,20,24,27,28,32)^.

Married women have greater marital and emotional stability, which leads to a better developed physiological and emotional status^(45)^, positively influencing self-efficacy levels. A prospective study conducted in Peru supports this relationship, describing that single mothers had an eight times greater risk of demonstrating a medium or low level of self-efficacy in breastfeeding when compared to those who were married or in a stable union^(28)^.

Furthermore, the lower level of maternal education negatively affects breastfeeding self-efficacy^(1,15,18,23-25)^. This proposition can be explained by the fact that verbal persuasion, one of the sources of self-efficacy listed by Bandura^(5)^, can be strengthened through educational interventions and have a better effect on mothers with higher levels of education, since they have a better ability to understand the information provided. Hydery *et al*.^(46)^ demonstrated that mothers with higher education, after watching educational videos, were significantly more likely to self-report healthy maternal behaviors, such as the likelihood of breastfeeding compared to mothers with less education, thus ratifying the relationship between educational level and adherence to breastfeeding, and, as a consequence, self-efficacy in breastfeeding.

Maternal occupation and breastfeeding self-efficacy also demonstrate a well-documented association in the literature, such that mothers who perform work activities outside the home have lower levels of self-efficacy^(1,15,22,27)^. Returning to work is considered one of the main factors that prevent continued breastfeeding^(22)^. A cross-sectional study conducted in Saudi Arabia with 1,689 postpartum women found that women who did not work outside the home were 1.6 times more likely to have high levels of breastfeeding self-efficacy compared to participants who worked^(1)^.

This context may be related to insufficient information regarding maternal rights as a breastfeeding worker or difficulty in accessing resources that facilitate milk extraction outside the home. Burns *et al*.^(47)^ confirm this relationship by demonstrating that mothers who are confident in their rights to extract milk and breastfeed in the workplace are more likely to maintain milk production after returning to work and achieve their breastfeeding goals.

Thus, for mothers who work outside the home and breastfeed, there is a particular demand for verbal persuasion actions to encourage and guide these women to continue breastfeeding and thus maintain good levels of self-efficacy. Therefore, increasing knowledge about their rights effectively improves these mothers’ confidence in their ability to maintain an adequate supply of breast milk and continue breastfeeding after returning to work^(47)^.

Maternal race and breastfeeding self-efficacy also show an interrelationship^(22)^. Breastfeeding in black mothers is influenced by cultural/ethnic and sociological domains due to a greater likelihood of being inserted in socially unfavorable and health contexts, with poor access to qualified care^(48)^. Such barriers in health care make breastfeeding difficult for black mothers over time^(49)^.

However, social support has been portrayed in the literature as a facilitator for successful breastfeeding among black mothers^(50)^. Breastfeeding support measures resulting from government initiatives, access to information and health care in the prenatal and postpartum periods, as well as social support from family, peers and the community through involvement and sharing of experiences, can promote the initiation and continuation of breastfeeding^(48)^, in addition to improving levels of maternal self-efficacy in breastfeeding, since social support represents an important source of vicarious experiences for breastfeeding mothers^(21)^.

It is worth noting that, in the preparation of the study, research was also identified that did not verify statistical associations between the aforementioned socioeconomic factors and breastfeeding self-efficacy in mothers in socioeconomic vulnerability^(16,19,21,26,29,38)^. The lack of significance between the associations may be due to sample size in some of the studies analyzed, the use of methodological designs with less precision in establishing causal relationships, as well as studies carried out with populations in developed countries with the interference of confounding factors in the results.

### Study limitations

Study limitations are related to specific aspects in scoping review operationalization, such as the absence of specific controlled vocabulary for socioeconomic vulnerability and the linguistic and scope restriction of the productions to only those that were available in full, which may have culminated in the reduced number of articles analyzed.

### Contributions to health

This SST presents relevant concepts and causal associations between the elements that permeate breastfeeding self-efficacy in mothers inserted in the context of socioeconomic vulnerability. The theory therefore constitutes a useful tool for the theoretical foundation of subsequent studies with this public and for the actions to be developed by professionals who work directly with this population.

## CONCLUSIONS

The breastfeeding Self-Efficacy Theory in mothers in socially vulnerable situations presents a more specific scope with greater practical applicability in this population. The present study identified certain socioeconomic factors that influence breastfeeding self-efficacy in the population analyzed, such as family income, maternal age, education, parity, maternal occupation, marital status, race, and social support.

In SST construction, the causal relationships between these factors and the sources of self-efficacy proposed by Bandura are explained. Among the individual factors, age and parity have influenced experiences of active mastery, while maternal occupation and education are related to verbal persuasion, marital status, with emotional and physiological state, and race, with vicarious experiences, when considering social support influenced by the ethnic context.

Contextual socioeconomic aspects have also affected self-efficacy sources so that a lower family income is directly related to the emotional and physiological state of mothers in socioeconomic vulnerability. Furthermore, social support acts positively on the levels of self-efficacy of mothers by influencing the vicarious experiences arising from the family and community support offered to them.

When considering the causal relationships between the SST elements, it can be inferred that providing guidance that is appropriate for the target audience and providing emotional and practical support are essential measures to mitigate certain effects of individual and contextual factors on levels of breastfeeding self-efficacy, enabling breastfeeding promotion in mothers in socioeconomic vulnerability.

## Data Availability

The research data are available within the article.
