# Supplementary material for: Breastfeeding self-efficacy in mothers in socioeconomic vulnerability: Situation-Specific Theory
Source: Rev Bras Enferm. 2025 Dec 8;78(5):e20240652. doi: 10.1590/0034-7167-2024-0652 (PMC12707769; doi:10.1590/0034-7167-2024-0652)
Supplement: Translation

# Translation: Autoeficácia em amamentar em mães em vulnerabilidade socioeconômica: Teoria de Situação Específica

**DOI:** 10.1590/0034-7167-2024-0652pt

**Authors:** Rebecca Camurça Torquato, Joana Maria Rocha Sales, Laysla de Oliveira Cavalcante Lima, Marcos Venícios de Oliveira Lopes, Viviane Martins da Silva, Lorena Pinheiro Barbosa

**Affiliations:** I Universidade Federal do Ceará. Fortaleza, Ceará, Brasil

**Keywords:** Autoeficácia, Teoria de Enfermagem, Mães, Aleitamento Materno, Fatores Socioeconômicos.

## Abstract

**Objetivos:**

desenvolver uma Teoria de Situação Específica baseada na Teoria da Autoeficácia aplicada às mães que amamentam em vulnerabilidade socioeconômica.

**Métodos:**

a Teoria de Situação Específica foi construída com base nos pressupostos de Callista Roy, estruturados em seis etapas: (1) definição da abordagem; (2) definição de modelos conceituais; (3) definição dos principais conceitos; (4) desenvolvimento do diagrama pictorial; (5) construção das proposições; (6) estabelecimento de relações causais e evidências para prática.

**Resultados:**

oito fatores socioeconômicos, como idade, paridade, ocupação materna, escolaridade, estado civil, raça, apoio social e renda familiar, interferem nas fontes de autoeficácia em mães em vulnerabilidade socioeconômica. Os elementos da Teoria de Situação Específica foram associados em um diagrama pictorial e em relações causais evidenciadas por seis proposições.

**Conclusões:**

conclui-se que a Teoria de Situação Específica pode proporcionar uma melhor compreensão das relações entre esses fatores com as fontes de Teoria de Autoeficácia em mães com vulnerabilidade socioeconômica.

## INTRODUÇÃO

As teorias de enfermagem favorecem o desenvolvimento teórico, contribuindo para um cuidado de qualidade e possibilitando que a enfermagem estruture seu campo profissional e científico^(1)^. Nessa perspectiva, teorias podem não ser aplicáveis a todas as estruturas sociais, conjuntura política ou período histórico^(2)^, sendo imprescindível o desenvolvimento de arcabouços teóricos que se adequem à população em questão e às suas necessidades.

As teorias podem ser definidas como o conjunto de pressupostos, princípios ou proposições que projetam uma visão sistemática de fenômenos extraídos da realidade, oferecendo a organização e estrutura do conhecimento^(3)^. Essas teorias podem variar de acordo com o nível de abrangência, de modo que as grandes teorias são amplas, enquanto a Teoria de Médio Alcance (TMA) e a Teoria de Situação Específica (TSE) são mais específicas^(4)^.

No contexto das grandes teorias, pode-se citar a Teoria da Autoeficácia de Bandura^(5)^. Esta teoria traz a autoeficácia como um conjunto de diferentes auto-crenças relacionadas a domínios de diversos funcionamentos^(5)^. Tais crenças de autoeficácia são elaboradas com base em quatro fontes essenciais de informação, a saber: experiências de domínio ativo, que funcionam como parâmetros de capacidade; experiências vicárias, que modificam a convicção de eficácia a partir da observação de modelos do outro; persuasão verbal, no qual a crença de autoeficácia é reforçada através da influência social de outrem; e estado fisiológico e afetivo, a partir do qual o indivíduo julga sua competência, força e fragilidade^(5)^.

As TMAs, por serem mais específicas, apresentam menos conceitos e envolvem um escopo mais limitado da realidade, com proposições concretas e definidas de modo operacional^(6)^. Grandes teorias e TMAs, contudo, apresentam limitações em retratar as diversidades e descrição de fenômenos específicos da enfermagem^(7)^. Desse modo, emergem, em 1999, as TSEs, que constituem teorias com foco em populações ou campos de prática específicos, inseridas em contextos sociais e históricos particulares, sendo consideradas mais práticas e apropriadas para pesquisa em grupos populacionais, quando comparadas às TMAs^(7)^.

Em 1999, a enfermeira Cindy-Lee Dennis, ao reconhecer a importância da grande Teoria da Autoeficácia e seguir seu entusiasmo sobre pesquisas envolvendo o aleitamento materno e as recomendações da Organização Mundial da Saúde sobre a promoção e estímulo deste para a saúde materno-infantil^(8)^, construiu um arcabouço teórico sobre a autoeficácia no contexto da amamentação. Nos seus escritos, a autora desenvolve argumentos que reforçam a aplicabilidade dos pressupostos teóricos de Bandura a uma realidade específica, como a amamentação, ao relacionar as quatro fontes de autoeficácia diante das especificidades de uma mulher que amamenta^(9)^.

No contexto da amamentação, há uma lacuna no desenvolvimento de TSE direcionada às mães inseridas em situações que desfavorecem o processo de aleitamento materno. Entre estas situações, ressalta-se a vulnerabilidade socioeconômica como uma condição em que os indivíduos afetados são menos propensos a enfrentar situações econômicas difíceis e acreditam não possuir direito a benefícios que possam contribuir para resguardar-lhes de tais privações^(10)^. Ao citar que estas condições influenciam a amamentação, questiona-se: como explicar o fenômeno da autoeficácia na amamentação em mães em situação de vulnerabilidade socioeconômica?

A construção de uma TSE para este público em específico pode contribuir para uma melhor compreensão de como a autoeficácia na amamentação se apresenta nesse contexto e, assim, dar aparato para o planejamento de um cuidado direcionado a essa mulher, visando à promoção do aleitamento materno e da saúde do binômio mãe-bebê.

## OBJETIVOS

Desenvolver uma TSE baseada na Teoria da Autoeficácia aplicada às mães que amamentam em vulnerabilidade socioeconômica.

## MÉTODOS

Este estudo seguiu seis etapas: definição da abordagem para a TSE; definição de modelos conceituais; definição dos principais conceitos; desenvolvimento de um diagrama pictorial; construção de proposições; estabelecimento de relações causais e evidências para prática^(11)^.

### Definição da abordagem para a Teoria de Situação Específica

Em busca de embasamento científico e reflexivo para a construção da TSE, utilizaram-se como base a Teoria de Autoeficácia de Bandura^(5)^ e os pressupostos teóricos de Callista Roy^(11)^, realizando-se revisão de escopo com protocolo cadastrado no *Open Science Framework* (https://doi.org/10.17605/OSF.IO/CFTA7). Esta foi relatada conforme *Preferred Reporting Items for Systematic Reviews and Meta-Analyses for Scoping Reviews* (PRISMA-ScR)^(12)^, seguindo as recomendações metodológicas propostas pelo JBI para revisões de escopo^(13)^.

A revisão foi realizada durante os meses de maio a junho de 2024. A questão norteadora teve como base o acrônimo População, Conceito, Contexto (PCC) indicado pelo JBI^(13)^, no qual “P” inclui mães que amamentam, “C”, autoeficácia em amamentar, e “C”, vulnerabilidade socioeconômica, o que gerou o questionamento a seguir: quais são os elementos e as relações que influenciam a autoeficácia em amamentar na população de mães que amamentam em vulnerabilidade socioeconômica? O Quadro 1 sintetiza as etapas realizadas.

**Quadro 1:** Questão de pesquisa e construção da estratégia de busca da revisão de escopo, Fortaleza, Ceará, Brasil, 2024

Questão de pesquisa	Quais são os elementos e as relações que influenciam a autoeficácia em amamentar na população de mães que amamentam em vulnerabilidade socioeconômica?		
	População	Conceito	Contexto
**Extração**	Mães que amamentam	Autoeficácia em amamentar	Vulnerabilidade socioeconômica
**Conversão**	Amamentação	Autoeficácia	Fatores socioeconômicos
**Combinação**	amamentação; aleitamento materno; aleitamento materno complementado; aleitamento materno exclusivo; *breastfed; breastfeeding; breast fed; breast feeding, exclusive; exclusive breast feeding; breastfeeding, exclusive; exclusive breastfeeding; complementary feeding; feeding, complementary; breast feeding education; breast feeding promotion; breast feeding support; breastfeeding education; breastfeeding promotion; breastfeeding support*	autoeficácia; *self efficacy; efficacy, self; self concept; concept, self; self; self awareness; self efficacy; self confrontation; self image; self perception; self rating; self representation; self concept*	fatores socioeconômicos; fatores econômicos e sociais; características socioeconômicas; iniquidade social; vulnerabilidade socioeconômica; *factor, socioeconomic; socioeconomic factors; factors, socioeconomic; social inequalities; socioeconomic factor; economic and social factors; social inequality; social and economic factors; socioeconomic vulnerability; socioeconomics; economic value of life; socioeconomic characteristics; characteristic, socioeconomic; health care, indigent; inequality, social; medical indigency; socioeconomic characteristic; indigent health care; social economic aspect; social economics; social-economic factor; socio-economic aspect; socio-economic factor; socio-economics; socioeconomic aspect; value of life; socioeconomics; social economic vulnerability; socio-economic vulnerabilit*y
**Construção**	*breastfed OR breastfeeding OR “breast fed” OR “breast feeding, exclusive” OR “exclusive breast feeding” OR “breastfeeding, exclusive” OR “exclusive breastfeeding” OR “complementary feeding” OR “feeding, complementary” OR “breast feeding education” OR “breast feeding promotion” OR “breast feeding support” OR “breastfeeding education” OR “breastfeeding promotion” OR “breastfeeding support” OR “breast feeding education”*	*“self efficacy” OR “efficacy, self” OR “self concept” OR “concept, self” OR self OR “self awareness” OR “self confrontation” OR “self efficacy” OR “self image” OR “self perception” OR “self rating” OR “self representation” OR selfconcept*	*“socioeconomic factors” OR “factor, socioeconomic” OR “socioeconomic factor” OR “economic and social factors” OR “factors, socioeconomic” OR “socioeconomic characteristics” OR “characteristic, socioeconomic” OR “socioeconomic characteristic” OR “social and economic factors” OR “social inequality” OR “inequality, social” OR “social inequalities” OR “socioeconomic vulnerability” OR socioeconomics OR “economic value of life” OR “health care, indigent” OR “indigent health care” OR “medical indigency” OR “social economic aspect” OR “social economics” OR “social-economic factor” OR “socio-economic aspect” OR “socio-economic factor” OR “socio-economics” OR “socioeconomic aspect” OR “value of life” OR “social economic vulnerability” OR “socio-economic vulnerability”*
**Estratégia de busca principal**	*((breastfed OR breastfeeding OR “breast fed” OR “breast feeding, exclusive” OR “exclusive breast feeding” OR “breastfeeding, exclusive” OR “exclusive breastfeeding” OR “complementary feeding” OR “feeding, complementary”) AND (“self efficacy” OR “efficacy, self”)) AND (“socioeconomic factors” OR “factor, socioeconomic” OR “socioeconomic factor” OR “economic and social factors” OR “factors, socioeconomic” OR “socioeconomic characteristics” OR “characteristic, socioeconomic” OR “socioeconomic characteristic” OR “social and economic factors” OR “social inequality” OR “inequality, social” OR “social inequalities” OR “socioeconomic vulnerability”)*		

*Fonte: adaptado de Araújo OWC, 2020^(14)^.*

Com base na questão norteadora, realizou-se uma busca nas bases de dados MEDLINE (Via PubMed), *Excerpta Medica Database* (Embase), Literatura Latino-Americana e do Caribe em Ciências da Saúde (LILACS), *Cumulative Index to Nursing and Allied Health Literature* via EBSCO (CINAHL), Scopus, *Web of Science* e *Cochrane Library*.

Foram incluídos estudos de todos os delineamentos metodológicos, disponibilizados na íntegra, em inglês, português e espanhol, sem restrição de data de publicação. Foram excluídos os artigos que não responderam à questão de pesquisa, bem como resenhas, editoriais, livros, capítulos, artigos de opinião, dissertações, teses e resumos de conferências em decorrência da flexibilidade dos critérios de avaliação dessas produções, e os artigos não disponibilizados na íntegra. As fases que envolveram o processo de seleção dos artigos analisados são descritas no diagrama PRISMA-ScR (Figura 1).

**Figura 1:** Diagrama *Preferred Reporting Items for Systematic Reviews and Meta-Analyses for Scoping Reviews* de busca e seleção dos artigos para a revisão de escopo, Fortaleza, Ceará, Brasil, 2024

Foi utilizado o *software* Rayyan para o gerenciamento das produções, e os artigos foram avaliados e selecionados a partir dos critérios de elegibilidade por dois revisores independentes e cegos. Eventuais divergências foram resolvidas por um terceiro revisor.

Os dados dos estudos selecionados foram extraídos e analisados a partir do preenchimento de um formulário de caracterização embasado nas diretrizes da JBI^(13)^ e estruturado com os seguintes dados: (1) características do estudo: título, ano, país, periódico, autores, tipo de estudo, objetivos; (2) características da população: tamanho da amostra, dados sociodemográficos, características gerais e de vulnerabilidade socioeconômica; (3) desfecho principal: autoeficácia em amamentar e fatores socioeconômicos relacionados.

### Definição dos modelos conceituais

Para a construção da TSE, foi usado o modelo conceitual da Teoria da Autoeficácia de Bandura^(5)^ para a construção dos conceitos, relações e pressupostos relatados neste estudo. Esta traz a autoeficácia como a confiança que o indivíduo apresenta na sua habilidade de desempenhar com sucesso certas atividades ou comportamentos. Essa convicção é construída por quatro principais fontes de autoeficácia, que são experiência pessoal, experiência vicária, persuasão verbal, e estado fisiológico e afetivo.

### Definição dos principais conceitos

De acordo com as evidências oriundas da revisão de escopo e os pressupostos da Teoria da Autoeficácia de Bandura^(5)^, realizou-se a seleção dos conceitos-chave para compor os elementos mínimos para a construção da TSE: autoeficácia na amamentação; experiência de domínio ativo; experiência vicária; persuasão verbal; estado fisiológico e afetivo; e vulnerabilidade socioeconômica.

### Desenvolvimento do diagrama pictorial

O diagrama pictorial deste estudo foi desenvolvido a partir das informações primárias e secundárias dos conceitos estruturantes da TSE, viabilizando o entendimento das relações causais.

### Construção de proposições

Para a explicitação das relações entre os conceitos dentro da TSE, foram elaboradas seis proposições para demonstrar as relações entre os fatores socioeconômicos e a autoeficácia em amamentar em mães em vulnerabilidade socioeconômica, estratificada nas quatros fontes de autoeficácia de Bandura.

### Estabelecimento de relações causais e evidências para prática

As relações de causalidade entre os elementos da TSE foram expressas dentro do diagrama pictorial, porém estas podem não ser visualizadas na sua totalidade. Desse modo, essas relações entre os elementos socioeconômicos e a autoeficácia em amamentar em mães em vulnerabilidade socioeconômica foram também descritas através das proposições para facilitar sua compreensão e uso da TSE na prática clínica.

## RESULTADOS

Os artigos selecionados para fornecer o embasamento teórico, juntamente à Teoria da Autoeficácia^(5)^, para o desenvolvimento da TSE, seus autores, ano de publicação e desenho do estudo, são descritos no Quadro 2.

**Quadro 2:** Descrição dos artigos incluídos na revisão de escopo, Fortaleza, Ceará, Brasil, 2024

Referências	Ano País	Título	Delineamento do estudo	Nível de evidência^*^
Siqueira *et al*.^(15)^	2023 Brasil	*Factors associated with breastfeeding self-efficacy in the immediate puerperium in a public maternity hospital*	Estudo transversal	Nível 6
Pinto *et al*.^(16)^	2021 Brasil	*Evaluation of breastfeeding self-effectiveness and its associated factors in puerperal women assisted at a public health system in Brazil*	Estudo transversal	Nível 6
Müller *et al*.^(17)^	2020 Brasil	*Self-efficacy and exclusive breastfeeding maintenance in the first months after childbirth*	Estudo coorte prospectivo	Nível 4
Santos *et al*.^(18)^	2020 Brasil	*Self-efficacy of breastfeeding in postpartum women assisted in a public maternity in northeastern Brazil*	Estudo transversal	Nível 6
Monteiro *et al*.^(19)^	2020 Brasil	*Breastfeeding self-efficacy in adult women and its relationship with exclusive maternal breastfeeding*	Estudo longitudinal e prospectivo	Nível 4
Silva *et al*.^(20)^	2018 Brasil	*Breastfeeding self-efficacy and interrelated factors*	Estudo transversal	Nível 6
Guimarães *et al*.^(21)^	2017 Brasil	*Factors related with breastfeeding self-efficacy immediate after birth in puerperal adolescents*	Estudo transversal	Nível 6
Santos *et al*.^(22)^	2022 Brasil	*Self-efficacy of puerperal women in breastfeeding: a longitudinal study*	Estudo longitudinal do tipo painel	Nível 4
Rodrigues *et al*.^(23)^	2015 Brasil	*Influence of sociodemographic and behavioral conditions on self-efficacy in breastfeeding: a cross-sectional study*	Estudo transversal	Nível 6
Uchôa *et al*.^(24)^	2014 Brasil	*Sociodemographic and obstetric history in maternal self-efficacy in nursing: a study in panel*	Estudo longitudinal do tipo painel	Nível 4
Margotti e Epifanio^(25)^	2014 Brasil	*Exclusive maternal breastfeeding and the Breastfeeding Self-efficacy Scale*	Estudo coorte descritiva e analítica	Nível 4
Nursan, Dilek e Sevin^(26)^	2014 Turquia	*Breastfeeding Self-efficacy of Mothers and the Affecting Factors*	Estudo descritivo	Nível 6
Dodt *et al*.^(27)^	2013 Brasil	*Influence of health education strategy mediated by a self-efficacy breastfeeding serial album*	Estudo quase-experimental	Nível 3
Quiroz *et al*.^(28)^	2013 Peru	*Lactancia materna exitosa en puérperas de menos de 48 horas en el Hospital de Apoyo María Auxiliadora*	Estudo transversal	Nível 6
Al-Thubaity *et al.* ^(1)^	2023 Arábia Saudita	*Determinants of High Breastfeeding Self-Efficacy among Nursing Mothers in Najran, Saudi Arabia*	Estudo transversal	Nível 6
Li *et al.* ^(29)^	2022 China	*Determinants of breastfeeding self-efficacy among postpartum women in rural China: A cross-sectional study*	Estudo transversal	Nível 6
Yang *et al*.^(30)^	2016 China	*Predictors of breastfeeding self-efficacy in the immediate postpartum period: A cross-sectional study*	Estudo transversal	Nível 6
Tavares *et al*.^(31)^	2010 Brasil	*Application of Breastfeeding Self-Efficacy Scale-Short Form to post-partum women in rooming-in care: a descriptive study*	Estudo transversal	Nível 6
Souza *et al*.^(32)^	2020 Brasil	Avaliação da autoeficácia na amamentação em puérperas	Estudo epidemiológico	Nível 6
Joshi *et al.* ^(33)^	2015 Estados Unidos da América	*Comparison of Socio-Demographic Characteristics of a Computer Based Breastfeeding Educational Intervention Among Rural Hispanic Women*	Estudo quase-experimental	Nível 3
Ku e Chow^(34)^	2010 China	*Factors influencing the practice of exclusive breastfeeding among Hong Kong Chinese women: a questionnaire survey*	Estudo transversal	Nível 6
Kamalifard *et al*.^(35)^	2019 Irã	*Relationship of Breastfeeding Self-Efficacy with Self-Esteem and General Health in Breastfeeding Mothers Referred to Health Centers of Falavarjan City-Iran, 2015*	Estudo transversal	Nível 6
Gökçeoğlu e Küçükoğlu^(36)^	2016 Turquia	*The relationship between insufficient milk perception and breastfeeding self-efficacy among Turkish mothers*	Estudo descritivo	Nível 6
Lima *et al*.^(37)^	2023 Brasil	Avaliação do uso de álbum seriado sobre amamentação como estratégia de intervenção educativa no puerpério	Estudo quase-experimental	Nível 3
Conde *et al*.^(38)^	2017 Brasil	*Breastfeeding self-efficacy and length of exclusive breastfeeding among adolescent mothers*	Estudo longitudinal prospectivo, observacional e analítico	Nível 4

* *Nível de evidência-Melnyk; Fineout-Overholt, 2023^(39)^.*

### Teoria de Situação Específica de autoeficácia em amamentar em mães em vulnerabilidade socioeconômica

O desenvolvimento da TSE foi baseado nos 25 artigos identificados na revisão, bem como nos pressupostos da Teoria da Autoeficácia. Quanto ao método, entre os artigos analisados, 13 eram estudos transversais, quatro, estudos longitudinais, três, estudos quase-experimentais, dois, estudos de coorte, dois, estudos descritivos, e um, estudo epidemiológico.

### Fatores socioeconômicos

Os fatores socioeconômicos identificados na revisão de escopo que podem influenciar a autoeficácia em amamentar foram idade materna, renda familiar, estado civil, número de filhos, escolaridade, ocupação, raça e apoio social.

O fator mais frequente foi o número de filhos, presente em dez artigos, seguido por idade materna e renda familiar, ambos relatados em nove estudos. Também são fatores relevantes para a autoeficácia para amamentar nesta população a escolaridade, citada em oito artigos, o estado civil, citado em sete artigos, o apoio social, citado em seis artigos, e ocupação, citado em cinco artigos. Além disso, observou-se que a raça, citada em apenas um estudo, também pode influenciar o objeto de estudo.

### Definição dos principais conceitos

A partir da revisão de escopo e das literaturas de base, foram desenvolvidos os principais conceitos para a TSE em questão, nomeadamente:

- Autoeficácia em amamentar: percepção da mãe a respeito da sua capacidade e habilidade de amamentar seu filho;
- Experiência de domínio ativo: vivência pessoal prévia em amamentação que auxilia a mãe na prática atual de aleitamento materno;
- Experiência vicária: observação de um indivíduo que vivenciou o processo de amamentação, podendo oferecer apoio à mãe ao compartilhar suas experiências;
- Persuasão verbal: transmissão do conhecimento através de orientações e incentivo por um indivíduo, que objetiva estimular a mãe a amamentar;
- Estado fisiológico e afetivo: condição emocional e de saúde na qual a mãe se encontra e que afeta, de forma positiva ou negativa, o seu processo de amamentação;
- Vulnerabilidade socioeconômica: situação na qual os fatores socioeconômicos afetam o processo de amamentar das mães, tornando-as mais suscetíveis a apresentar dificuldades em estabelecer o aleitamento materno e manter níveis adequados de autoeficácia em amamentar.

### Desenvolvimento do diagrama pictorial

Após o desenvolvimento dos principais conceitos, foi realizada a construção do diagrama pictorial para auxiliar na compreensão da TSE (Figura 2).

**Figura 2:** Pictograma representativo das relações causais da Teoria de Situação Específica para autoeficácia em amamentar em mães em vulnerabilidade socioeconômica, Fortaleza, Ceará, Brasil, 2024

### Construção de proposições

De acordo com Fawcett^(40)^, proposições são princípios verídicos sem a necessidade de comprovação. Para a TSE deste estudo, foram elaboradas as seguintes proposições:

A menor idade materna e, como consequência, maior chance de primiparidade, são fatores reforçadores e precipitantes, respectivamente, que podem proporcionar baixos níveis de autoeficácia em amamentar, visto que essas mulheres, em geral, não possuem experiência de domínio ativo com a amamentação;

Ser solteira é um fator precipitante para baixos níveis de autoeficácia em amamentar, pois a ausência de um companheiro afeta o estado fisiológico e afetivo da mulher;

Possuir baixa renda é um fator incapacitante que pode intervir negativamente nos níveis de autoeficácia em amamentar, por afetar o estado emocional e fisiológico da mãe inserida em contexto de apreensão decorrente de recursos financeiros escassos;

Manter atividade laboral fora do lar, considerado um fator incapacitante, pode estar relacionado a menores níveis de autoeficácia em amamentar, visto que as mães possuem acesso limitado a informações oriundas de persuasão verbal suficientes para manter a amamentação após o retorno ao trabalho;

A baixa escolaridade é um fator reforçador que pode prejudicar a compreensão das mães acerca das informações advindas de atividades de persuasão verbal, o que desfavorece sua autoeficácia em amamentar;

A raça materna, considerada um fator predisponente, pode influenciar os níveis de autoeficácia em amamentar, visto que etnia e apoio social, classificado como fatores precipitantes, viabilizam o compartilhamento de experiências vicárias, estimulando a continuidade da amamentação.

### Estabelecimento de relações causais e evidências para prática

As relações causais entre os fatores socioeconômicos e a autoeficácia em amamentar estão descritas de forma detalhada no diagrama pictorial e nas proposições. Os elementos influenciam os níveis de autoeficácia em amamentar, de modo que: apoio social, estado civil e paridade são considerados fatores precipitantes; renda e ocupação são consideradas fatores incapacitantes; idade e escolaridade são consideradas fatores reforçadores; e raça é considerada fator predisponente.

## DISCUSSÃO

A Teoria de Autoeficácia em amamentar em mães em vulnerabilidade socioeconômica explana conceitos essenciais para a construção do conhecimento a respeito desse fenômeno, além de elencar os fatores socioeconômicos intervenientes na autoeficácia apresentada pelas mães nesta situação específica. Relações causais entre os principais conceitos da TSE construída serão explanados de acordo com as evidências extraídas da literatura vigente.

O número de filhos, a idade materna e a renda são os fatores socioeconômicos mais frequentemente associados à autoeficácia em amamentar na clientela analisada. A primiparidade é um fator que predispõe a uma reduzida autoeficácia em amamentação^(1,15,17,18,22,23,25,28,30)^. Essa proposição se relaciona às experiências pessoais prévias em aleitamento materno comuns em mães multíparas e ausente nas primíparas, influenciando uma das fontes de autoeficácia apontadas por Bandura^(5)^ e ressaltada por Dennis^(9)^ como fundamental para alta autoeficácia em amamentar^(1)^.

A maioria das evidências também expõe que a idade materna mais avançada está associada a níveis elevados de autoeficácia em amamentar^(15,18,22,27,28,31)^. A idade materna e paridade são fatores, por sua vez, interrelacionados. Mulheres que têm o primeiro filho em idade jovem podem ter mais nascimentos ao longo da vida^(41)^, o que reflete em experiências anteriores em amamentação, aumentando os níveis de autoeficácia em amamentar nessas mães^(1,42)^.

Apesar de estar bem estabelecida essa relação, determinados estudos conduzidos no Brasil^(17,18)^ e na China^(34)^ evidenciam que estratos etários inferiores têm relação com autoeficácia em amamentar mais alta em virtude do apoio advindo da mãe/sogra, atuando como fontes de experiência vicária para mães mais jovens^(21)^.

A renda familiar e a autoeficácia em amamentar em mães em vulnerabilidade socioeconômica também apresentam relação diretamente proporcional, e isso pôde ser observado em diversos estudos desta revisão^(15,20,22,24,27,31,34)^. Essa relação pode ser esclarecida pelo fato de que a renda familiar afeta diretamente o estado fisiológico e afetivo da mãe, uma das fontes de autoeficácia propostas por Bandura, à medida que a nutriz se preocupa com a situação familiar e isso pode afetar seu processo de lactação e, consequentemente, a amamentação e a autoeficácia para colocá-la em prática.

Rodrigues *et al*.^(23)^ e Kamalifard *et al*.^(35)^ validam essa proposição ao apontar que a presença de quatro ou mais pessoas coabitando em um mesmo domicílio com fonte de renda única e o desemprego dos cônjuges são fatores associados a um nível reduzido de autoeficácia materna em amamentar, uma vez que essas condições conduzem a situações econômicas desfavoráveis, afetando o estado fisiológico e emocional da mulher, o que acarreta o declínio da condição de saúde geral concomitante à autoeficácia em amamentação.

Ainda em relação à renda familiar e sua interferência na amamentação, estudo realizado por Uchôa *et al*.^(24)^ identificou que a presença de serviços básicos de esgotamento e fornecimento de água influencia positivamente o nível de autoeficácia em amamentar em mães em vulnerabilidade socioeconômica. A baixa cobertura desses serviços e a inacessibilidade à coleta de resíduos sólidos podem ser fatores prejudiciais associados à autopercepção de saúde^(43)^, afetando o estado fisiológico e emocional da mãe, e retroalimentando o *status* de baixa autoeficácia em amamentar. Apesar da importância, países de baixo ou médio rendimento carecem de gestão dos resíduos e acesso ao saneamento e fornecimento de água, alertando para a necessidade de uma melhor cobertura, de modo a produzir efeitos substancialmente benéficos à saúde^(44)^, bem como à autoeficácia em amamentar das mães.

Nas mães em vulnerabilidade socioeconômica, o estado civil também se mostrou diretamente associado à autoeficácia em amamentar. O apoio de maridos ou parceiros também é considerado um preditor de autoeficácia em amamentação^(30)^, de modo que mães casadas e em união consensual apresentaram maiores níveis de autoeficácia em amamentar^(15,20,24,27,28,32)^.

Mulheres casadas têm maior estabilidade conjugal e emocional, o que conduz a um *status* fisiológico e emocional melhor desenvolvido^(45)^, influenciando positivamente os níveis de autoeficácia. Estudo prospectivo realizado no Peru corrobora essa relação, ao descrever que as mães solteiras apresentaram um risco oito vezes maior de demonstrar nível médio ou baixo de autoeficácia em amamentar quando comparadas àquelas casadas ou em união estável^(28)^.

Ademais, o menor nível de escolaridade materna afeta negativamente a autoeficácia em amamentação^(1,15,18,23-25)^. Tal proposição pode ser explicada pelo fato de que a persuasão verbal, uma das fontes de autoeficácia elencada por Bandura^(5)^, poder ser fortalecida através de intervenções educativas e surtir melhor efeito em mães com escolaridade mais elevada, visto que essas têm uma melhor capacidade de compreender as informações fornecidas. Hydery *et al*.^(46)^ demonstraram que mães com ensino superior, após assistirem a vídeos educativos, foram significativamente mais propensas a auto-referirem comportamentos maternos saudáveis, como a probabilidade de amamentar, em comparação com mães com menor escolaridade, ratificando, assim, a relação entre nível educacional e adesão à amamentação, e, como consequência, a autoeficácia em amamentar.

A ocupação materna e a autoeficácia em amamentar também demonstram associação bem documentada na literatura, de modo que mães que desempenham atividades laborais extradomiciliares apresentam menores níveis de autoeficácia^(1,15,22,27)^. O retorno ao trabalho é considerado um dos principais fatores impeditivos para a continuidade da amamentação^(22)^. Estudo transversal, realizado na Arábia Saudita com 1.689 puérperas, constatou que as mulheres que não mantinham atividades laborais fora do lar eram 1,6 vezes mais propícias a terem altos níveis de autoeficácia em amamentar, se comparadas às participantes que trabalhavam^(1)^.

Esse contexto pode estar relacionado a informações insuficientes quanto aos direitos maternos enquanto trabalhadora que amamenta ou dificuldade de acesso a recursos que facilitem a extração de leite fora do domicílio. Burns *et al.* ^(47)^ confirmam essa relação, ao demonstrar que mães que confiam nos seus direitos de extrair o leite e amamentar no ambiente de trabalho têm maior chance de manter a produção láctea após o regresso às suas atividades laborais e atingir seus objetivos de amamentação.

Desse modo, para as mães que exercem atividade laboral fora do domicílio e amamentam, há uma demanda particular de ações de persuasão verbal para estimular e orientar tais mulheres a continuar no processo de amamentação e, assim, permanecer com bons níveis de autoeficácia. Portanto, aumentar o conhecimento sobre seus direitos melhora eficazmente a confiança dessas mães na sua capacidade de manter um aporte adequado de leite materno e dar continuidade à amamentação após o regresso ao trabalho^(47)^.

A raça materna e a autoeficácia em amamentar também manifestam uma interrelação^(22)^. A amamentação em mães negras apresenta influência de domínios cultural/étnico e sociológico decorrente de uma maior probabilidade de estarem inseridas em contextos socialmente desfavoráveis, e de saúde, com acesso precário à assistência qualificada^(48)^. Tais barreiras nos cuidados de saúde dificultam a amamentação por mães negras no decorrer do tempo^(49)^.

Contudo, o apoio social tem sido retratado na literatura como um facilitador para o sucesso da amamentação entre mães negras^(50)^. Medidas de apoio à amamentação decorrentes de iniciativas governamentais, acesso à informação e cuidados de saúde nos períodos pré-natal e pós-parto, bem como apoio social da família, dos pares e da comunidade por meio do envolvimento e compartilhamento de experiências, podem promover o início e a continuação da amamentação^(48)^, além de melhorar os níveis de autoeficácia materna em amamentar, uma vez que o apoio social representa uma importante fonte de experiências vicárias para as mães que amamentam^(21)^.

Vale salientar que, na elaboração do estudo, também foram identificadas pesquisas que não verificaram associações estatísticas entre os fatores socioeconômicos supracitados e a autoeficácia em amamentar nas mães em vulnerabilidade socioeconômica^(16,19,21,26,29,38)^. A ausência de significância entre as associações pode ser decorrente do tamanho amostral em alguns dos estudos analisados, uso de delineamentos metodológicos com menor precisão no estabelecimento das relações causais, bem como estudos realizados com populações em países desenvolvidos com a interferência de fatores confundidores nos resultados.

### Limitações do estudo

As limitações do estudo estão relacionadas a aspectos específicos na operacionalização da revisão de escopo, como ausência do vocabulário controlado específico para vulnerabilidade socioeconômica e restrição linguística e de abrangência das produções para apenas aquelas que estavam disponíveis na íntegra, o que pode ter culminado na reduzida quantidade de artigos analisados.

### Contribuição para a área da saúde

Esta TSE traz conceitos e associações causais relevantes entre os elementos que permeiam a autoeficácia em amamentar em mães inseridas no contexto de vulnerabilidade socioeconômica. A teoria constitui, portanto, em ferramenta útil para a fundamentação teórica de estudos posteriores com esse público e para as ações a serem desenvolvidas pelos profissionais que atuam diretamente com essa população.

## CONCLUSÕES

A Teoria de Autoeficácia em amamentar em mães em vulnerabilidade socioeconômica apresenta uma abrangência mais específica com maior aplicabilidade prática nessa população. O presente estudo identificou determinados fatores socioeconômicos que influenciam a autoeficácia em amamentar no público analisado, tais como renda familiar, idade materna, escolaridade, paridade, ocupação materna, estado civil, raça e apoio social.

Na construção da TSE, são explanadas as relações causais entre esses fatores e as fontes de autoeficácia propostas por Bandura. Entre os fatores individuais, a idade e a paridade têm influenciado experiências de domínio ativo, ao passo que a ocupação materna e a escolaridade têm relação com a persuasão verbal, o estado civil, com o estado fisiológico e emocional, e a raça, com experiências vicárias, ao considerar o apoio social influenciado pelo contexto étnico.

Aspectos socioeconômicos contextuais também têm afetado as fontes de autoeficácia, de modo que uma menor renda familiar tem relação direta com o estado fisiológico e emocional das mães em vulnerabilidade socioeconômica. Além disso, o apoio social atua positivamente nos níveis de autoeficácia das mães com a influência nas experiências vicárias advindas do suporte familiar e comunitário oferecido a elas.

Ao considerar as relações causais entre os elementos da TSE, infere-se que o fornecimento de orientações com adequabilidade ao público em questão e o suporte de apoio emocional e prático são medidas essenciais para a atenuação de determinados efeitos dos fatores individuais e contextuais nos níveis de autoeficácia em amamentar, possibilitando a promoção do aleitamento materno em mães em vulnerabilidade socioeconômica.

## Data Availability

Os dados da pesquisa estão disponíveis no corpo do artigo.
